# An Unusual Presentation of Kawasaki Disease: Gallbladder Hydrops and Acute Cholestatic Hepatitis

**DOI:** 10.1155/2018/4930234

**Published:** 2018-07-03

**Authors:** B. O. Kılıç, Ş. Baysun, T. C. Gökşen, İ. Akınbingöl, Z. Arslan

**Affiliations:** Departments of Pediatrics, Faculty of Medicine, TOBB University of Economics and Technology, Ankara, Turkey

## Abstract

Kawasaki disease is the most common vasculitis of childhood. In its classical form, at least four of five diagnostic criteria including cervical lymphadenopathy (1.5 cm or more), nonsuppurative conjunctivitis, intraoral mucosal changes, edema in hands and feet, and maculopapular rash are required with prolonged fever over 5 days. Atypical cases which are different from the classical type or incomplete cases which does not include all the diagnostic criteria can be seen. The typical Kawasaki disease is a self-limiting disease with fever lasting for an average of two weeks. In such patients who have not been diagnosed and whose treatment has been delayed, coronary artery aneurysm, myocardial depression, arrhythmia, and vascular complications may increase morbidity and mortality. We would like to present a rare case of an atypical patient with gallbladder hydrops and acute cholestatic hepatitis.

## 1. Case

**Figure 1:**
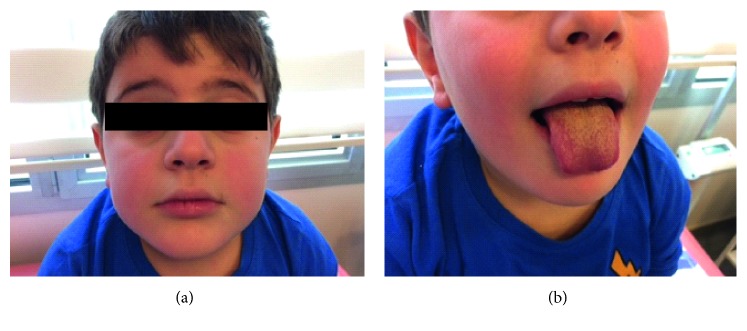
Nonpurulent conjunctivitis, malar rash, and mucosal changes on the lip and on the tongue.

A 7-year-old boy presented due to ongoing fever and abdominal pain for 5 days. He had vomiting once and watery stool twice on the same day. On physical examination, he had fever of 39°, restlessness, bilateral nonpurulent conjuctival hyperemia, redness of the lip and tongue, polymorphic rash in the face and trunk, and obvious abdominal tenderness in the right upper quadrant (Figure 1).

In laboratory examination, hemoglobin was 13 g/dl, leukocyte was 15700/mm^3^, thrombocyte was 93000/mm^3^, CRP was 171 mg/dl (0–5 mg/dl), erytrocyte sedimentation rate was 75 mm/h, aspartate aminotransferase was 109 U/L (15–50 U/L), alanine aminotransferase was 202 U/L (10–50 U/L), total bilirubin was 3.49 mg/dl (0.3–1.35 mg/dl), direct biluribine was 3.42 mg/dl (0.05–0.5 mg/dl) and sodium was 126 meq/l (130–150 meq/L). Adenovirus was negative in nasal swab and stool. Stool microscopy was normal. Serology tests were negative for hepatitis A, B, and C. Abdominal ultrasonography showed acute cholangitis/cholecystitis, thickening of the gallbladder wall, hydrops, and intrahepatic bile duct stasis.

Echocardiography (ECHO) showed minimal pericardial effusion, and mild mitral and tricuspid regurgitation in the left ventricle. Troponin I value (HST) was found to be 22.3 ng/L (normal value < 0.2).

The patient was considered to have incomplete Kawasaki disease, and he was given a single dose of immunuglobulin (IVIG) with a 12-hour intravenous infusion of 2 g/kg and acethylsalicylic acid (ASA) of 50 mg/kg/day divided into 4 doses.

Cultures were taken, and treatment with ceftriaxone 80* *mg/kg for enteric fever and cholecystitis was initiated. The next day, antibiotic was stopped because of negative results of microbial cultures. Forty-eight hours after the patient's fever returned to normal, aspirin was reduced to only one dose of 3–5 mg/kg.

The patient's platelet count increased to 676,000/mm^3^ in the second week. Coronary artery involvement was not observed in the first echocardiography. Troponin I level also fell below 1.5 ng/L. In the third week, sedimentation and CRP values returned to normal and ASA treatment was terminated.

## 2. Discussion

In addition to fever as a classical symptom of Kawasaki disease, our patient had nonpurulent conjunctivitis, redness of lip and tongue, and polymorph rash, with no coronary artery involvement at the ECHO. Febrile cholestatic hepatitis with gallblader hydrops was thought to be the symptoms for atypical Kawasaki disease. Diarrhea, vomiting, abdominal pain, hepatic dysfunction, and bile duct hydrops had been reported at different rates among nonspecific symptoms even though they are not the part of the diagnostic criteria [1].

In a case-control study, approximately 30% of the 280 patients had moderate transaminase elevation and occasionally obstructive jaundice since gallblader hydrops was observed [2]. In a cohort study from Italy with 219 kawasaki disease cases, incomplete Kawasaki presentation was seen in 9 of 10 children with severe abdominal discomfort, and it was shown that the gastrointestinal system symptoms were not recognized early in Kawasaki disease [3]. However, acute cholestasis and gallblader hydrops were also reported as atypical baseline findings [4].

In another study, Kawasaki disease was detected in 6% of the patients with fever and abdominal pain [5].

In a Kawasaki disease series with 35 cases, reported from Turkey, acute cholestasis was not reported as a baseline finding [6]. However, in another study of 23 patients, hydrops of gallblader was reported in 1 case and transaminase elevation in 7 cases [7]. Kaman et al. also reported 2 cases of atypical kawasaki disease with acute febrile jaundice [8].

Vasculitis-associated inflammation and obstruction in the liver and gallbladder are thought to be the cause of increased transaminase levels and cholestasis. It is also known that the sodium level below 135 meq/l increases the risk for coronary artery disease, and the risk is higher in atypical cases than in classical cases. In addition to the atypical clinical presentation of our patient, hyponatremia and high transaminases increased the risk for complication developed that is why IVIG treatment was started on the sixth day of fever, and no complication was observed.

With this case report, we would like to remind that gallblader hydrops and cholestasis can be nonclassical early findings of Kawasaki disease, and if considered, early IVIG treatment can rapidly improve the clinical findings and prevent complications.
